# Who Is Still Playing Pokémon Go? A Web-Based Survey

**DOI:** 10.2196/games.7197

**Published:** 2017-04-05

**Authors:** Peter Rasche, Anna Schlomann, Alexander Mertens

**Affiliations:** ^1^ Institute of Industrial Engineering and Ergonomics Department of Mechanical Engineering RWTH Aachen University Aachen Germany; ^2^ Doctoral Programme GROW “Gerontological Research On Well-Being” University of Cologne Cologne Germany

**Keywords:** games, recreational, mobile apps, cell phones, Pokémon Go

## Abstract

**Background:**

Poor physical activity is one of the major health care problems in Western civilizations. Various digital gadgets aiming to increase physical activity, such as activity trackers or fitness apps, have been introduced over recent years. The newest products are serious games that incorporate real-life physical activity into their game concept. Recent studies have shown that such games increase the physical activity of their users over the short term.

**Objective:**

In this study, we investigated the motivational effects of the digital game “Pokémon Go” leading to continued use or abandonment of the game. The aim of the study was to determine aspects that motivate individuals to play augmented reality exergames and how this motivation can be used to strengthen the initial interest in physical activity.

**Methods:**

A total of 199 participants completed an open self-selected Web-based survey. On the basis of their self-indicated assignment to one of three predefined user groups (active, former, and nonuser of Pokémon Go), participants answered various questions regarding game experience, physical activity, motivation, and personality as measured by the Big Five Inventory.

**Results:**

In total, 81 active, 56 former, and 62 nonusers of Pokémon Go were recruited. When asked about the times they perform physical activity, active users stated that they were less physically active in general than former and nonusers. However, based on a subjective rating, active users were more motivated to be physically active due to playing Pokémon Go. Motivational aspects differed for active and former users, whereas fan status was the same within both groups. Active users are more motivated by features directly related to Pokémon, such as catching all possible Pokémon and reaching higher levels, whereas former users stress the importance of general game quality, such as better augmented reality and more challenges in the game. Personality did not affect whether a person started to play Pokémon Go nor their abandonment of the game.

**Conclusions:**

The results show various motivating elements that should be incorporated into augmented reality exergames based on the game Pokémon Go. We identified different user types for whom different features of the game contribute to maintained motivation or abandonment. Our results show aspects that augmented reality exergame designers should keep in mind to encourage individuals to start playing their game and facilitate long-term user engagement, resulting in a greater interest in physical activity.

## Introduction

Daily physical activity is one of the leading strategies for fighting global mortality [1]. Although organizations such as the World Health Organization constantly promote the value of physical activity, the trend is the opposite [1]. In Germany, physical activity is continually decreasing within the population. This trend is present in all age groups. In 2016, more than half of the entire German population performed an inadequate amount of physical activity per day [2]. As a result, health care costs rise, and the probability of secondary diseases such as high blood pressure rises, too. Thus, solutions for motivating individuals to perform physical activity on a daily basis are more essential than ever.

Recent attempts to encourage individuals to perform physical activity include activity trackers and fitness apps, which have turned mobile phones into a personal measuring instrument to document daily physical activity [3]. By documenting and defining a certain daily goal in this manner, the user is encouraged to reconsider his or her lifestyle and incorporate more physical activity into their everyday life [4,5]. Further, elements such as challenges, badges, or rank lists with family, friends, or a community are used to motivate the users of an activity tracker to perform a healthy amount of daily physical activity [6-8]. Recently, an additional trend occurred in augmented reality exergames, also referred to as urban exergames [9], which incorporate real-life physical activity into their game concept. Urban exergames are characterized by a set of criteria: the player is required to be physically active, the game is played in an urban environment, it runs on mobile phones, and makes use of the built-in mobile phone sensors [9]. Medical and public health communities have discussed the potential of these games with regard to their influence on higher levels of sustainable physical activity to achieve health benefits [10].

The most successful game in this category in 2016 was Pokémon Go. It is an augmented reality game for iOS and Android released in July 2016. The game is based on fictional creatures called Pokémon (ref. to Pocket Monsters), which first came on the scene in the 1990s and were merchandized in video games, card games, movies, television series, comic books, toys, etc. The aim of Pokémon Go is to seek, hunt, and collect a variety of different Pokémon as in previous video games. However, instead of launching just another video game, Niantic, the developer of Pokémon Go, combined the geocaching concept with augmented reality mechanics. This augmented reality feature embeds two-dimensionally animated Pokémon in real-world images captured by the mobile phone camera. Users have to explore their real-world neighborhood to search for and hunt Pokémon. The individual Pokédex of every user provides an overview of which Pokémon have already been found and caught. The central element of the game is to catch and collect all the different Pokémon. Other features include training Pokémon and fighting against the Pokémon of other users in battle arenas. By performing various physical activities in Pokémon Go, the users gain experience points that are required to reach higher levels.

The launch of Pokémon Go led to hype all over the world. Large numbers of users met on streets and in public places [11]. Despite the relatively short time since its release, there is initial research on Pokémon Go. Current studies have mainly investigated the effect of Pokémon Go on physical activity [12,13]. Althoff et al showed that persons who are more interested in Pokémon Go, measured by search queries, are more active than those who are less interested [13]. Similar research also exists for other video games requiring physical activity, such as Wii Fit or Kinect Sports [14]. Going beyond objectively measured physical activity, it is, however, also relevant to consider the effects of playing Pokémon Go on further domains, such as social cognitive factors including the users’ self-perception, behavioral intentions, and motivational aspects. Social and game-related correlates such as attitudes toward gaming and habits have been shown to influence active gaming among adolescents [15]. One study reported benefits and negative effects for children playing Pokémon Go [16]. Pokémon Go is a good research object due to its high number of users. Additionally, Pokémon Go includes several of the popular gamification tactics as described by Cugelman [8]. The game offers a clear theme by integrating Pokémon into the real world. The story is also quite easy to tell, with the overall goal being to collect all Pokémon and become the best Pokémon trainer [16]. Furthermore, the game offers clear goals (catch ‘em all), challenges (hatch an egg), levels (experience levels), and allocation points (Pokéstops and arenas), and it shows the progress of the user, provides feedback, and rewards experience points. A badge is awarded each time an egg hatches, and the game leaders are shown at the top of each arena they are actually the best in.

To the best of our knowledge, we are the first to investigate the aspects of the motivation to start and continue playing an augmented reality exergame like Pokémon Go in the general population.

In this study, we investigated the influence of personality and various game functions on physical activity and motivation to start playing Pokémon Go as well as on motivation to continue playing the game or quitting. We performed a Web-based survey questioning motivation to start, continue, and quit, as well as personality based on the Big Five Inventory [17,18].

In summary, our main research questions are

1. How long do users play Pokémon Go?
2. What are the aspects motivating people to start playing Pokémon Go?
3. What are the aspects motivating users to continue playing Pokémon Go?
4. What are the aspects motivating users to quit playing Pokémon Go?
5. Are there any subjectively perceived effects of playing this augmented reality exergame on physical activity?
6. Which type of users engages with Pokémon Go?
7. How can these effects be transferred to other augmented reality exergames?

Our study provides guidance on how to initially get individuals engaged in augmented reality exergames and how to facilitate long-term user engagement.

## Methods

### Design

An open, self-selected, Web-based survey was designed to investigate the aforementioned research questions. The survey was designed in German and provided for German-speaking internet users. A Web-based survey was used as it is a suitable way to reach individuals with particular characteristics or interests, that is, the group of potential game users, in a short period of time without any limitations on physical space [19-21].

On the basis of the research questions, the main purpose of the survey was to collect data about three different user groups that we would like to compare. The three predefined user groups we wanted to identify and compare were individuals who actively play Pokémon Go, individuals who had played it, and others who had never played Pokémon Go before. To identify these three groups, users were asked to state in an initial question to which of these three groups they belong. On the basis of their answer, further thematic blocks were questioned, including physical activity and motivational aspects.

To differentiate between active and former users of Pokémon Go, more detailed questions about the duration of use and level reached were asked.

#### Measuring Physical Activity

On the basis of the idea of Godin and Shephard, physical activity was examined subjectively in one question asking how many times per week a person spends at least 30 min performing physical activity that causes sweat [22]. Due to the idea that individuals who do not regularly perform physical activity might also respond to our survey, we also included the answers “several times per month,” “once a month,” “rarely,” and “never” [23]. Active and former users also answered questions on whether playing Pokémon Go affects their subjective interest in physical activity and whether they think they perform more physical activity as a result of playing Pokémon Go.

#### Measuring Motivation

Active and former users answered questions about motivational aspects. These referred to the initial motivation to start playing Pokémon Go, the motivation to continue playing, and to missing functions in the game. Former users were also asked for the reasons they stopped playing and about additional features they would like to see incorporated into the game. All of these questions included an open-ended text field. In this context, we also investigated possible motivating effects by peers and co-users and possible interdependencies of playing the game with the user’s personal network.

#### Measuring Personality

To determine which type of user engages with Pokémon Go, the Big Five Inventory was applied. The concept of the Big Five Inventory is quite old but nevertheless it is a practical tool in characterizing individuals. The Big Five dimensions of personality are calculated based on 10 questions rated on a 5-point Likert scale (1=“not correct,” 5=“fully correct”) [17,18]. The Big Five dimensions are extraversion, agreeableness, conscientiousness, neuroticism, and openness. In this study, we used the Big Five Inventory to investigate whether the five dimensions of personality can be used to differentiate types of Pokémon Go users as well as persons with no intention of playing this game. If there are differences, game designers could bear this in mind and cater their games to certain personalities.

### Data Collection

Data were collected between October 26 and November 20, 2016. The questionnaire was programmed and made available on a website hosted using the Unipark software [24]. The survey was introduced as a study examining the effects of modern digital games on health care systems (see Multimedia Appendix 1).

All participants were informed about the duration of the survey, data storage, and the leading investigator. Each participant decided to take part in this survey voluntarily by following the designated link to the survey. No incentives were offered for participation.

The survey was tested properly by 2 independent examiners with regard to wording and technical functionality. The survey included 42 items for all 3 investigated user groups, distributed over 7 different pages. Participants were able to review their entries per page before moving on.

### Recruitment

The survey was addressed to the general population with access to the Internet in Germany. No exclusion criteria or screening questionnaires were applied.

We applied different channels of recruitment to reach a broad range of potential participants for this open survey. The sampling procedure was nonprobabilistic and respondents were selected based on their voluntary willingness to participate [19]. The Web-based survey was promoted by a Facebook advertisement targeting persons aged between 14 and 99 years, who had indicated on Facebook that they were interested in physical activity and well-being, entertainment electronics, or Pokémon Go. This method of recruitment was chosen because the probability that the participants are younger and familiar with social media is quite high. Furthermore, this open Web-based survey is an observational study targeting participants who play or have played Pokémon Go. Recruitment via social media, therefore, seems to be a suitable approach [20].

The advertisement itself used text similar to the text presented on the introduction page for the Web-based survey (see Multimedia Appendix 1). In addition, the weblink to the Web-based survey was posted in one private Facebook group (“RWTH Aachen University”) and on a Facebook fan page called “Pokémon Go Deutschland.” The former group is frequently used by students of RWTH Aachen University and consists of 17,221 members at the time of recruitment. The Facebook fan page “Pokémon Go Deutschland” was followed by about Pokémon Go fans at the time of recruitment. In total, 12,516 individuals saw the link to our survey presented in their newsfeed or group on Facebook. The weblink to the survey was also posted in the German Web community “Pokémon Go Forum,” which has 2456 members. Finally, the link to our open Web-based survey was distributed in a mailing list for students at the University of Cologne, Germany. In all cases, the recruitment was based on the same text as shown in Multimedia Appendix 1.

In total, n=345 unique individuals visited the website of our Web-based survey. The identification of different individuals was performed using the Unipark software based on Internet Protocol (IP) address and cookie function. N=88 of these 345 visitors never started the survey. N=58 discontinued completing the survey. In total, 199 visitors finally participated in the survey and completed the whole questionnaire. Of those, 53 were recruited through Facebook, 62 via the Pokémon Go forum, and 12 via email. For 72 participants, the channel of recruitment was unknown. The participation rate was thus 74.4% and the completion rate 60.9%. The average duration of completing the survey was 10 min 52.96 s with a median of 9 min 2 s.

### Statistical Analysis

Data were analyzed using the SPSS statistics software, version SPSS 22 (IBM). Several one-way analyses of variance (ANOVA) and multivariate analyses of variance (MANOVA) were conducted at a significance level of .05. To compare active and former users, we also calculated *t* tests for independent samples and chi-square statistics both at a significance level of .05.

### Ethics Statement

The Ethics Committee at RWTH Aachen Faculty of Medicine authorized this study and its ethical and legal implications in its statement EK236/16 in mid-2016.

## Results

### Participants

Depending on the answers to the first question in the survey, participants were divided into three groups (Table 1): active users of Pokémon Go (n=81), former users of Pokémon Go (n=56), and nonusers of Pokémon Go (n=62).

**Table 1:** Participant demographics by user group.

Demography		Participants: Pokémon Go users		
		Active (n=81)	Former (n=56)	Non (n=62)
**Age (in years)**				
	Minimum	19	15	15
	Maximum	60	66	85
	Mean (SD)	34.9 (9.8)	25.6 (8.4)	38.8 (19.6)
**Gender**				
	Male	54	34	32
	Female	27	22	30
**Education**				
	School pupil	0	3	3
	Low level	4	0	0
	Average level	16	1	6
	High level	55	51	51
	Other	6	1	2
**Environment**				
	Urban area	59	47	52
	Rural area	22	9	10
**Household**				
	Partner	22	19	15
	Family	39	15	24
	Shared flat	8	14	4
	Single	13	9	17
Duration of use (in months), Mean (SD)		3.9 (0.8)	1.6 (1.3)	-^a^
Level, Mean (SD)		27.0 (3.9)	14.9 (7.1)	-^a^

^a^No data available for nonusers.

### Physical Activity

**Figure 1:**
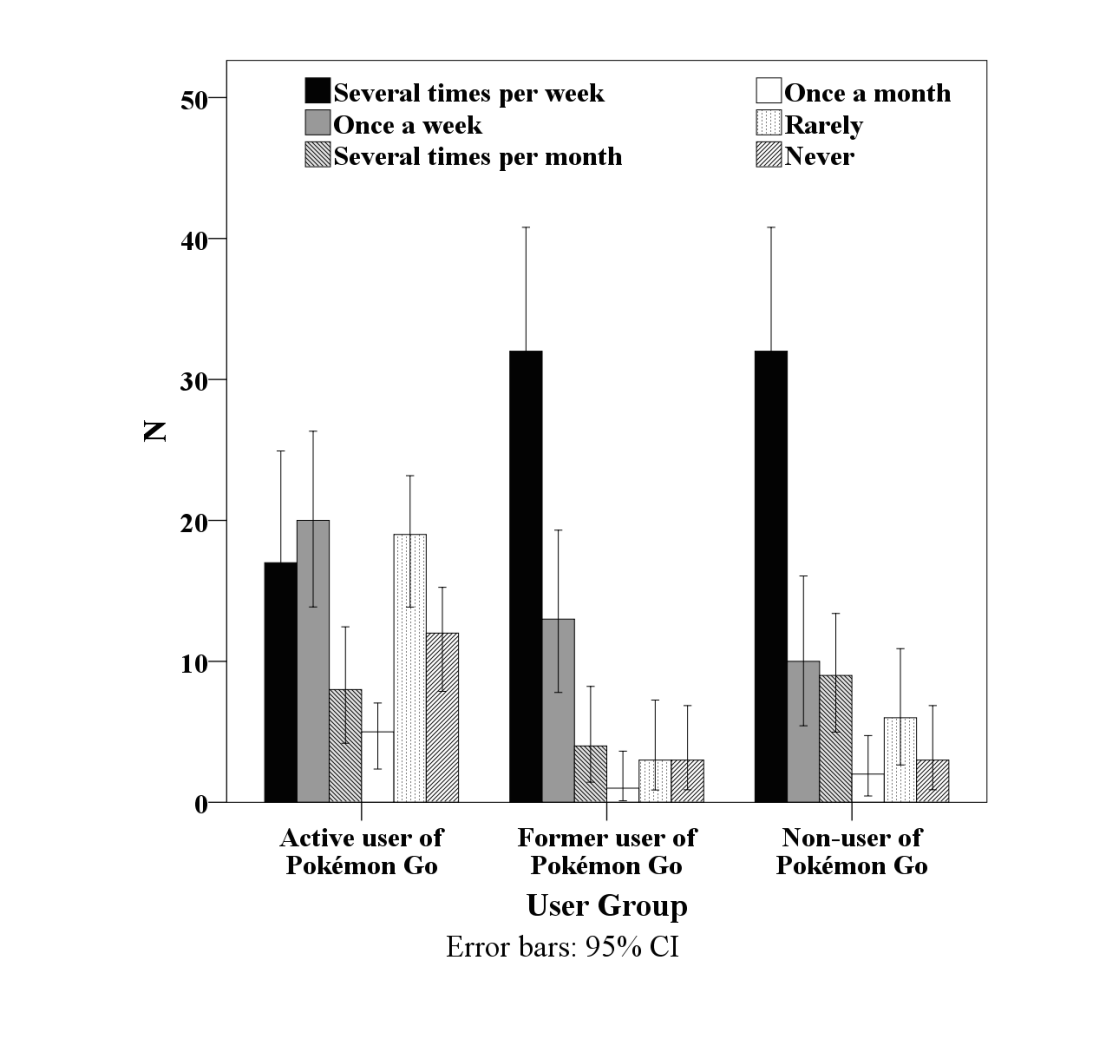
Self-evaluation on how often physical activity is performed.

Figure 1 shows the results for the question about self-rated physical activity for all participants. A descriptive analysis reveals that former as well as nonusers of Pokémon Go tend to perform physical activity “several times a week,” whereas the group of active users is spread over the whole range of performing physical activity “several times per week” to “never.” A univariate analysis of variance revealed significant differences in the physical activity behavior between the three groups (*F*_2,196_=14.359, *P*<.001).

**Figure 2:**
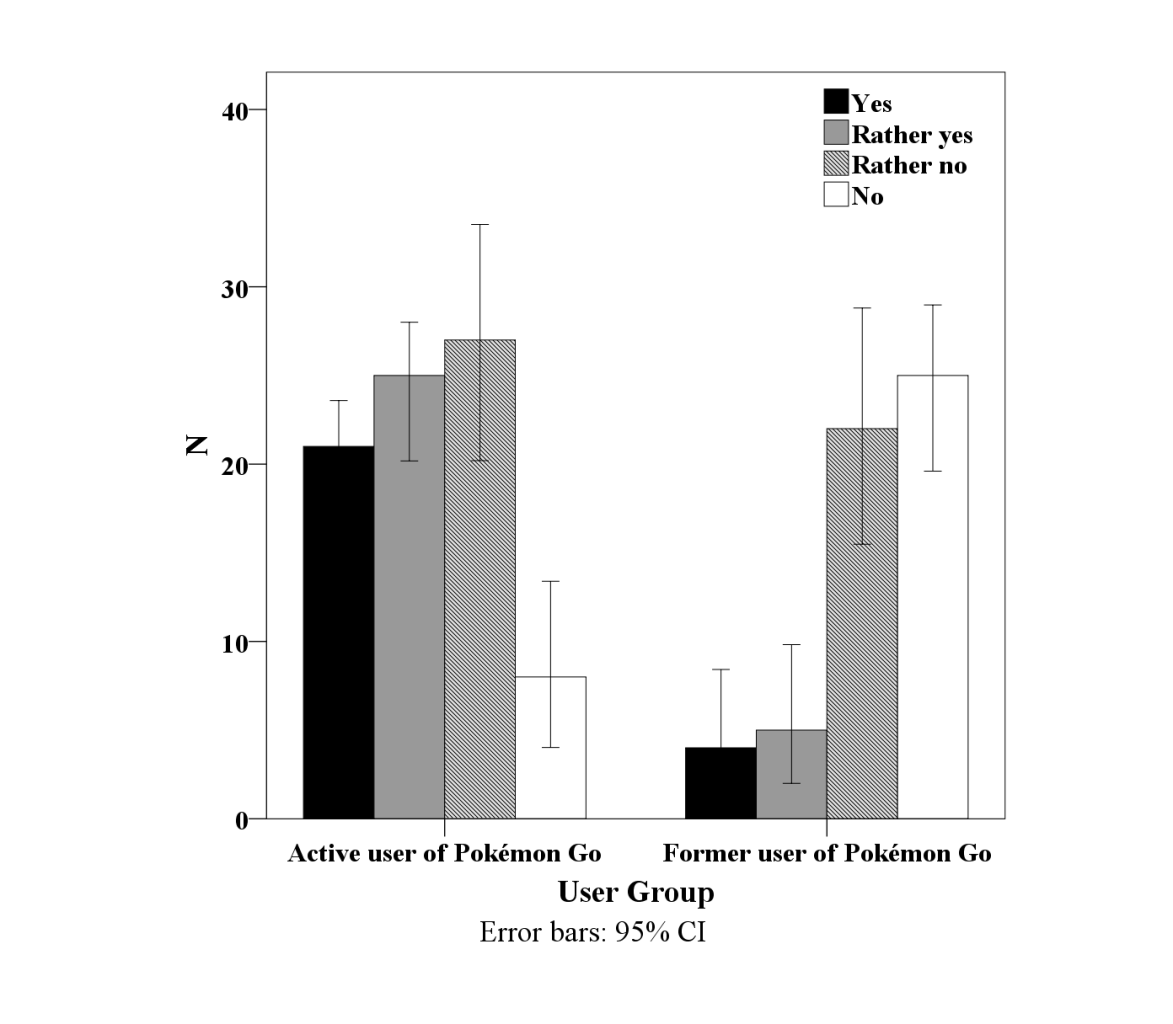
Subjective impression of whether Pokémon Go influenced users’ interest in performing physical activity.

Participants were asked about whether they believed Pokémon Go increased their interest in performing physical activity. As Figure 2 shows, active users had the impression that Pokémon Go increased their interest in physical activity. Former users did not believe that Pokémon Go increased their interest in performing physical activity. A univariate analysis of variance revealed significant differences in interest between active and former users (*F*_1,135_ = 33.818, *P*<.001).

Participants were also asked whether they had the impression that they were performing more or less physical activity since playing Pokémon Go.

**Figure 3:**
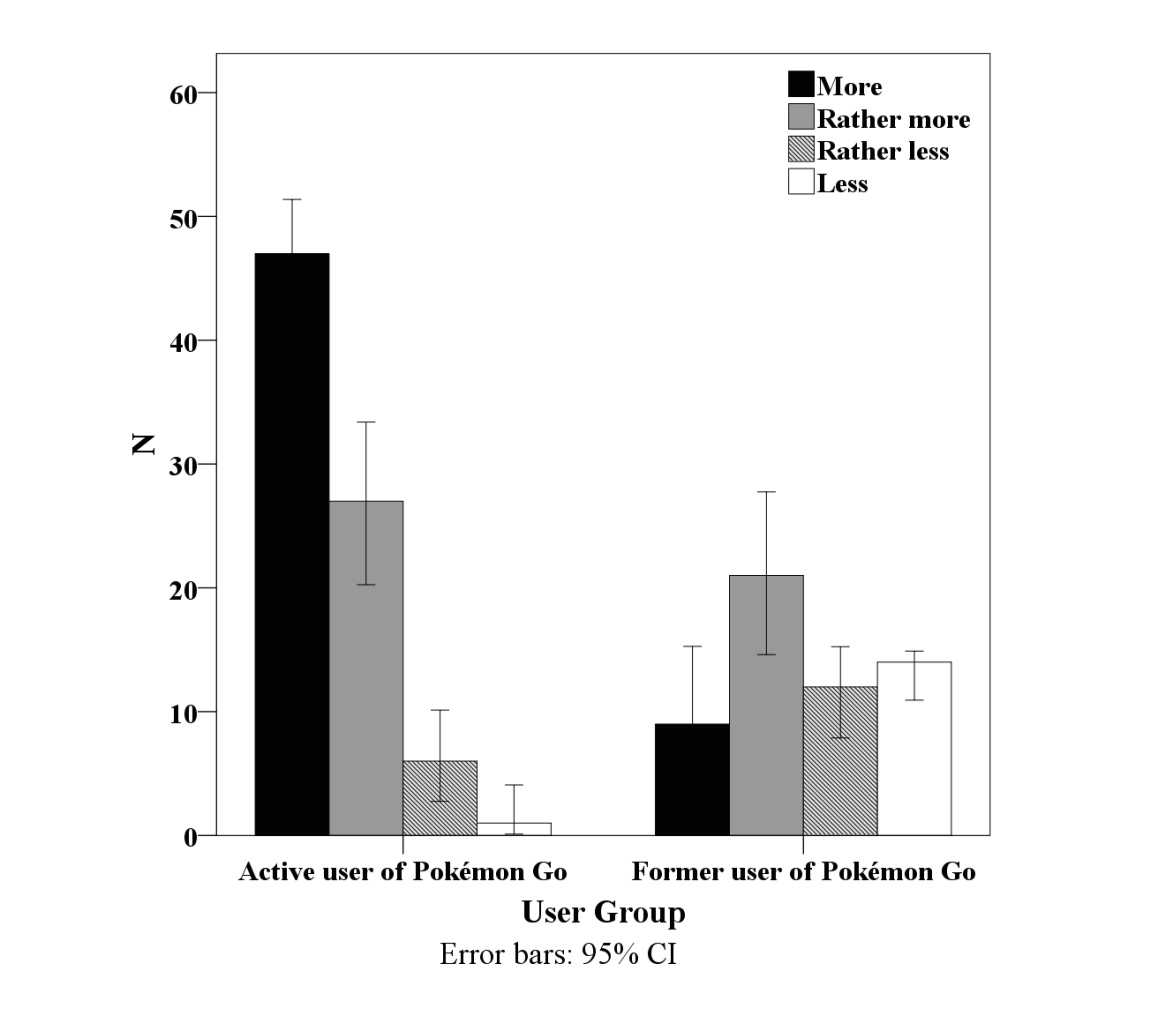
Subjective impression of whether more physical activity was performed than usual due to playing Pokémon Go.

The majority of active Pokémon Go users (47/81, 58%) stated that they performed more physical activity than before playing this game. Answers among the former Pokémon Go users were more divergent, as shown in Figure 3. A one-way analysis of variance with “user group” as the between-subject factor revealed a significant difference between the two groups (*F*_1,135_ = 48.833, *P*<.001).

### Motivation

The following section reports the findings related to motivational aspects. We focus especially on the motivation to start, continue playing, and quit the game.

#### Motivation to Start Playing the Game

On average, active and former users reported two reasons to start playing Pokémon Go (*M*_active_ 1.9 (SD 1.1), *M*_former_ 2.0 (SD 1.1)). There was no difference in the number of reasons given by the two groups (*t*_135_ = −0.53, *P*=.60). Table 2 provides an overview of the different aspects of motivation to start playing. Both groups mentioned curiosity most frequently. Being a POKÉMON fan, media reports, and reports from friends were also important sources of motivation for both groups (see Table 2). The only significant difference between active and former users of Pokémon Go occurred for the item “Being fascinated by the augmented reality function”; former users reported this reason more often than active users (χ^2^_1_ = 5.8, *P*=.02).

**Table 2:** Motivation to start playing Pokémon Go (multiple answers allowed).

Motivation to start playing	Pokémon Go users		Significance	*P* value
	Active (n=81)	Former (n=56)		
Mean of number of reasons (SD)	1.9 (1.1)	2.0 (1.1)	*t*_135_=−0.53	.60
Curiosity, n (%)	55 (68)	36 (64)	χ^2^_1_=0.2	.66
Being a Pokémon fan, n (%)	32 (40)	21 (38)	χ^2^_1_=0.1	.81
Media reports, n (%)	23 (28)	15 (27)	χ^2^_1_=0.0	.83
Reports from friends, n (%)	22 (27)	22 (39)	χ^2^_1_=2.2	.13
Everybody around me plays it, n (%)	11 (14)	5 (9)	χ^2^_1_=0.7	.41
Being fascinated by the augmented reality function, n (%)	5 (6)	11 (20)	χ^2^_1_=5.8	.02
Combining fun and physical activity^a^, n (%)	3 (4)	0 (0)	χ^2^_1_=2.1	.15
Game for traveling^a^, n (%)	2 (3)	0 (0)	χ^2^_1_=1.4	.24
Nostalgia^a^, n (%)	1 (1)	2 (4)	χ^2^_1_=0.8	.36

^a^Answers to open-ended questions; coded for analysis.

#### Motivation to Continue Playing the Game

Participants were asked whether reaching the next level motivated them to continue playing (10-point scale: 1=did not motivate at all, 10 = highly motivated). The mean value for the group of active users was 7.1 points (SD 2.1); the mean value for the group of former users was 5.4 points (SD 2.6). The two groups differ significantly (*t*_101_= 4.07, *P*<.001). In open-ended questions, participants were able to indicate which other aspects of the game besides reaching the next level motivated them to continue playing. Table 3 reports the reasons given. Active users were more motivated by the aim of completing the Pokédex (χ^2^_1_=26.9, *P*<.001) and reported more fun and curiosity while playing (χ^2^_1_=4.6, *P*=.03). There was no significant difference for any other reason given, but active users reported more reasons on average (*t*_131_=4.65, *P*<.001; see Table 3).

**Table 3:** Motivation to continue playing the game (multiple answers allowed).

Motivation to continue playing	Pokémon Go users		Significance		*P* value	
	Active (n=81)	Former (n=56)				
Mean of number of reasons (SD)	1.1 (0.8)	0.5 (0.7)		*t*_131_=4.65		<.001
Completing the Pokédex^a^, n (%)	33 (41)	1 (2)		χ^2^_1_=26.9		<.001
Fun or curiosity or recreation^a^, n (%)	12 (15)	3 (4)		χ^2^_1_=4.6		.03
Finding new or rare Pokémon^a^, n (%)	9 (11)	4 (7)		χ^2^_1_=0.6		.44
Catching strong Pokémon or being the best^a^, n (%)	8 (10)	10 (18)		χ^2^_1_=1.9		.17
Joint activities with family and friends^a^, n (%)	5 (6)	3 (5)		χ^2^_1_=0.0		.84
Being active or outside^a^, n (%)	5 (6)	2 (4)		χ^2^_1_=0.5		.50
Updates or new generations^a^, n (%)	4 (5)	0 (0)		χ^2^_1_=2.9		.09
Higher levels^a^, n (%)	3 (4)	1 (2)		χ^2^_1_=0.4		.51
Incubating eggs^a^, n (%)	2 (3)	1 (2)		χ^2^_1_=0.1		.79
Fighting in arenas^a^, n (%)	2 (3)	0 (0)		χ^2^_1_=1.4		.24
Nostalgia^a^, n (%)	2 (3)	0 (0)		χ^2^_1_=1.4		.24

^a^Answers to open-ended questions; coded for analysis.

Beyond motivational aspects directly related to the game, we also analyzed whether there is motivation due to social interaction. On the basis of active and former users’ self-reports, social contacts did not grow or decline through playing Pokémon Go. In total, 90% (73/81) of active users and 95% (53/56) of former users reported that their group of friends remained constant. There was no difference between the two groups (χ^2^_1_=0.9, *P*=.34). We found significant differences between active and former users for the question about how often Pokémon Go is a relevant topic in conversations in meetings with friends and family. In all, 50% (28/56) of former users never talk about Pokémon Go, and a further 39% (22/56) seldom talk about it; 11% (6/56) indicated that they talk about it often. For the active users, 11% (9/81) never, 56% (45/81) seldom, 20% (16/81) often, 5% (4/81) almost always, and 9% (7/81) always talk about Pokémon Go when meeting friends (χ^2^_4_=29.6, *P*<.001). We also asked users to rate the probability of recommending the game to others on a scale from 1 to 10. Active users would recommend the game more often than former users (*M*_active_ 7.43 (SD 2.2); *M*_former_ 4.21 (SD 2.3), *t*_135_=8.39, *P*<.001).

To examine aspects that motivate users to continue playing as a whole, we also asked about missing functions in the game. The missing functions differ for active and former users. For active users, a higher number of Pokéstops and more arenas are more important. Former users mention the possibility of exchanging Pokémon and better augmented-reality functions significantly more often than active users (see Table 4). For both groups, more Pokémon in the neighborhood and the possibility of exchanging Pokémon are further important features that are currently missing in Pokémon Go. Only 4% (3/81) of active and 4% (2/56) of former users said that there are no missing functions. On average, 2.8 missing functions were mentioned in both groups (SD 1.6 and 1.5).

**Table 4:** Missing functions in Pokémon Go (multiple answers allowed).

Missing functions	Pokémon Go users		Significance		*P* value	
	Active (n=81)	Former (n=56)				
Mean of number of functions (SD)	2.8 (1.6)	2.8 (1.5)		*t*_135_=0.084		.93
No missing functions, n (%)	3 (4)	2 (4)		χ^2^_1_=0.0		.97
More Pokémon in my neighborhood, n (%)	47 (58)	35 (63)		χ^2^_1_=0.3		.60
Exchanging Pokémon, n (%)	45 (56)	42 (75)		χ^2^_1_=5.4		.02
Direct fights against others, n (%)	44 (54)	38 (68)		χ^2^_1_=2.5		.11
More Pokéstops, n (%)	36 (44)	12 (21)		χ^2^_1_=7.7		.01
More updates, n (%)	31 (38)	14 (25)		χ^2^_1_=2.6		.10
More arenas, n (%)	21 (26)	6 (11)		χ^2^_1_=4.8		.03
Better augmented reality, n (%)	2 (3)	8 (14)		χ^2^_1_=6.8		.01

**Table 5:** Participants’ personality dimensions by user group.

Big five dimensions	Pokémon Go users		
	Active Mean (SD)	Former Mean (SD)	Non Mean (SD)
Extraversion (points)	3.2 ( 1.0)	3.5 (1.0)	3.4 (1.0)
Agreeableness (points)	2.9 (0.8)	3.0 (0.7)	3.1 (0.8)
Conscientiousness (points)	3.4 (0.8)	3.4 (1.0)	3.7 (1.0)
Neuroticism (points)	2.8 (1.0)	3.0 (1.0)	2.7 (0.8)
Openness (points)	3.5 (1.0)	3.5 (1.1)	3.5 (1.0)

Former users were also asked for reasons that would make them start playing again. The most frequently reported reasons were an increase in the range of functions (12/56, 21%) and options for interaction with other users (18/56, 32%). Further answers related to technical features such as more stable servers and a lower battery consumption (7/56, 13%) and rendering the game more interesting by incorporating new challenges and more tactical game elements (6/56, 11%).

#### Motivation to Quit

Individuals who were categorized as former users of Pokémon Go were asked for their reasons for quitting the game. The most frequently reported reasons were boredom (32/56, 57%), being disappointed (13/56, 23%), difficulties in reaching higher levels (16/56, 29%), and technical problems (10/56, 18%). Other points of criticism were related to missing components in the game itself, such as too few Pokémon (10/56, 18%), Pokéstops (5/56, 9%), and arenas (3/56, 5%) or a lack of co-users (4/56, 7%). Some former users also said that their general interest in the game had waned (6/56, 11%) or that they did not have the time to play (5/56, 9%). On average, 1.93 reasons were mentioned (SD 1.2).

### Personality

Mean values for the five personality dimensions within the different user groups are shown in Table 5.

A MANOVA was performed to investigate the effect of the between-subject factor “user group” on the different factors of the Big Five Inventory. Using Pillai’s trace, there was no significant effect of “user group” on the five factors of the Big Five Inventory (*V*=.059, *F*_10,386_=1.165, *P*=0.31). Also, separate univariate ANOVAs revealed no significant effects of the between-subject factor “user group” for the separate factors of the Big Five Inventory (.17< *P*<.99).

## Discussion

### Principal Findings

The potential of mobile phone apps to increase physical activity and thereby contribute to better health is intensively being discussed these days [25]. Going beyond classical fitness apps and wearable devices such as activity trackers, initial studies investigating the effects of augmented reality exergames such as Pokémon Go on physical activity are available [8,9]. The focus of this study lies on the motivation for starting to play, continuing to play, and quitting this game.

This study presents results of an open Web-based survey. The sample is divided into three groups (active (N=81), former (N=56), and nonusers of Pokémon Go (N=62)). An investigation of self-reported physical activity showed that the percentage of persons who rarely or never perform physical activity with a duration of at least 30 min while perspiring is higher in the group of active users than in the group of former or nonusers. Examining motivation to start this game showed that curiosity and being a fan of Pokémon were the most frequently mentioned aspects. It is interesting that the group of former users mentioned interest in the augmented reality technology significantly more often as motivation to start playing Pokémon Go.

Regarding the motivation to continue playing, this study revealed that the group of active users is motivated by aspects directly related to the aim of completing the Pokédex and reaching higher levels in the game. The group of former users was significantly less motivated to continue playing by aspects such as reaching the next level. Their efforts were much more competitive. They were motivated by catching strong Pokémon and becoming the best. Aspects relating to social interaction such as having fun, being outside, and spending time with family and friends while playing the game also motivated them to continue.

Former users were asked about aspects that motivated them to quit the game. The most frequently mentioned aspects were boredom and disappointment. Besides these aspects, missing social interaction was also mentioned again, such as, for example, exchanging Pokémon or fighting directly against each other. This was also highlighted by active users as a missing function in the game. Finally, the augmented reality function was criticized as being not realistic enough. However, if this issue were resolved, former users would be willing to give the game a second chance.

Our investigation regarding differences in personality within the different groups studied revealed no results. The use of this game is independent of personality.

### Gamification

The augmented reality exergame Pokémon Go employs a range of gamification elements. The effectiveness of gamification has been discussed in different areas of application as well, for example, to support the self-management of chronic diseases [26]. The crucial question in this context is whether gamification can contribute to long-term user engagement since only then is it reasonable to assume positive effects on physical activity and health as described in other works [8,9]. In our survey, we found that by no means was everyone who started to use the game motivated to continue playing in the long term. This phenomenon has also been shown for other apps triggering healthy behavior [27] and for the use of activity trackers [28]. However, using and quitting the use of mobile technologies and wearables is a complex process and not only caused by mere dissatisfaction [29]. In the following section, the gamification elements are discussed in detail with regard to their contribution to motivation according to the three main topics: starting to play, continuing to play, and quitting playing.

#### Motivation to Start Playing the Game

Curiosity, being a Pokémon fan, and the augmented reality function were the most frequently mentioned reasons to start playing Pokémon Go. Media and download reports also showed that telling the right story or theme in combination with a new technology, in this case the little-known augmented reality function, could motivate thousands to start playing the game [16]. Other games in this context also need to find the right triggers to create curiosity and get people to start playing. The use of cartoon characters generally seems to be a promising approach that has also been shown to affect children’s food preferences when placed on food packaging [30]. The augmented reality function was a motivating factor, especially for the former players. This leads to the conclusion that new functions or technologies could encourage the start of use, but it takes more to facilitate long-term use.

#### Motivation to Continue Playing the Game

In previous studies, rewards, competitions, and fun elements have been judged as important elements leading to enjoyable experiences in game-playing [7,26]. App design and specific app features are also crucial for the users’ long-term engagement [31]. These factors have also been shown in the present study. With regard to the motivation to continue playing, we found differences related to the classical concept of levels [32]. Active users were motivated by reaching the next level, whereas former users reported being more motivated by catching strong or rare Pokémon. Social interaction in real life regarding Pokémon Go, such as spending time with family and friends, was also much more motivating for former users than for active ones. It is therefore of great importance for user’s long-term engagement to consider individualized preferences. It furthermore seems important to integrate the game as far as possible into the users’ real lives, especially if an augmented reality function is used. Motivation for continuing playing the game could thus be strengthened.

#### Motivation to Quit

We should not ignore the fact that fairly high numbers of users have quit playing the game after a short period of use. One of the reasons mentioned was boredom. Within our study, the duration of playing Pokémon Go is even shorter than the average time of use for activity trackers, as reported in Ledger and McCaffrey [28]. Our results show that a strong interest in the theme of the game (in this case Pokémon Go) could prevent people from quitting it. In the event of such interest, reaching the next level was also experienced as motivating for users.

Missing social interaction functions within the game was a further reason for quitting the game. Social interaction and support were already important features demanded by users in earlier active games designed for the Nintendo Wii or Microsoft Xbox Kinect [33]. Tateno et al stated that Pokémon Go could be useful for increasing social contact outside the game itself [34]. Our study indicates that no social interaction outside the game arises as a result of just playing the game. Therefore, it is essential to include social interaction within the gameplay and the topic of the game. In the case of the investigated game, Pokémon Go, desired social interactions embedded in the game included direct fighting against each other without visiting an arena and swapping Pokémon among each other. Both aspects relate to highly realistic gameplay as Pokémon trainers could exchange Pokémon and fight against each other in the Pokémon books.

#### Personality

Analysis using the Big Five Inventory among users revealed no indication of significant differences among users playing Pokémon Go or quitting it once it was played. The comparison of the Big Five Inventory with participants indicating no interest in playing Pokémon Go showed no differences.

### Transfer of Knowledge

All in all, the design and the incorporated gamification functions of Pokémon Go are suitable for different types of users. Although the initial motivation to start was the same for active as well as former users, the motivation to continue playing was mainly linked to social interaction. Social interaction was the main function identified as missing in this game and, furthermore, it was identified as the function motivating long-term use. If a user is not fully immersed in the theme, social interaction and especially social rewards are the elements motivating users less interested in the theme of the game to continue playing. This is independent of a certain personality or user type. Therefore, augmented reality exergames should incorporate functions that support social interaction among users as well as between users and their friends and family.

### Limitations

This study has several limitations related to its methodological design as well as the reported results. The open Web-based study was not representative due to regional recruiting via Facebook. Although the users of Facebook are adequate in terms of representative population characteristics, a bias is still possible [21]. A bias in recruitment might lead to differences for the groups in the Big Five Inventory as well as in age and education. We also included only self-reports and no objective measures for physical activity. In terms of distinguishing individuals with high physical activity from those with medium or low physical activity, this is still a limitation [23]. Furthermore, this study was conducted 14 weeks after the initial start of Pokémon Go in Germany. Therefore, our study could only reveal initial motivational aspects for active and former users due to the lack of a long-term perspective. Nevertheless, we were able to show that users already existed who had quit playing the game after quite a short duration of use. To examine the motivational structures in more detail, longitudinal studies are needed to obtain a deeper insight into the mechanisms, as conducted, for example, in the context of activity trackers [6,28]. A qualitative follow-up could also be useful to track motivation over time.

Due to the open Web-based recruitment, no inferences can be made about the usage rated and sociodemographical distribution of the general user group of Pokémon Go, especially as this Web-based survey was conducted in German. Finally, it must be noted that we are unable to answer the question about how much time has passed since former users quit playing the game. Although we know how long the average duration of use is, it might be interesting to determine whether a former user was an early adopter or late adopter of this game.

### Conclusions

In an exploratory approach, we ascertained motivational structures in the context of serious mobile games that can serve as the basis for future work. To the best of our knowledge, this is the first study explicitly investigating the motivation of active and former Pokémon Go users to use and stop using the game. We were able to determine aspects motivating users to start playing Pokémon Go as well as reasons to quit the game. Further insights into how to maintain long-term user engagement have been revealed and compared with recent studies in the field of serious games and activity trackers.

## Appendix

Multimedia Appendix 1 Introduction text of survey (German/English).

## Abbreviations

ANOVA: one-way analyses of variance
MANOVA: multivariate analyses of variance
