# Supplementary material for: Who Is Still Playing Pokémon Go? A Web-Based Survey
Source: JMIR Serious Games. 2017 Apr 5;5(2):e7. doi: 10.2196/games.7197 (PMC5399220; doi:10.2196/games.7197)
Supplement: Multimedia Appendix 1 [file games_v5i2e7_app1.pdf]

Introduction text of survey (German)

'Vielen Dank, dass Sie sich entschieden haben, an unserer Umfrage zum Thema "Pokémon Go" teilzunehmen.
Im Folgenden werden wir Ihnen einige Fragen stellen, in denen wir von Ihnen erfahren möchten, ob Sie Pokémon Go kennen und ob Sie dieses Spiel bereits gespielt haben oder noch aktiv spielen. Dies hilft uns dabei, zu untersuchen, welchen Einfluss Spiele wie Pokémon Go auf die Gesundheitsversorgung haben können.
Die Beantwortung der Fragen nimmt maximal 10 Minuten Ihrer Zeit in Anspruch.Wichtig bei der Beantwortung der Fragen ist, dass es keine richtige oder falsche Antwort gibt. Antworten Sie entsprechend Ihrer Ideen und Überzeugung.
Wir bedanken uns für Ihre Unterstüzung und freuen uns auf Ihre Rückmeldung!
Anna Schlomann und Peter Rasche

Diese Umfrage wird im Rahmen eines Forschungsprojekt vom Bundesministerium für Bildung und Forschung durchgeführt (www.tech4age.de). Ihre Antworten werden im Sinne guter wissenschaftlicher Praxis anonym erhoben und dienen lediglich der wissenschaftlichen Forschung. Ihre Daten werden nicht an Firmen oder Dritte weitergegeben. Die Datenerhebung erfolgt im Einklang mit dem Verfahrensverzeichnis 10-15 beaufsichtigt durch den Datenschutzbeauftragten der RWTH Aachen University.'

Introduction text of survey (English translation)

'Thank you for choosing to participate in our Pokémon Go survey.
Below we will ask you some questions in which we would like to know from you whether you are familiar with Pokémon Go and whether you have already played this game or are still actively playing. This will help us investigate the impact of games like Pokémon Go on healthcare.
Answering the questions will approximately take a maximum of 10 minutes of your time. It is important to note that there are no right or wrong answers when answering these questions. Gives answers according to your ideas and beliefs.
We thank you for your support and look forward to your feedback!
Anna Schlomann and Peter Rasche

This survey is conducted within the framework of a research project by the Federal Ministry of Education and Research (www.tech4age.de). The answers are anonymous in line with good scientific practice and are only used for scientific research. Your data will not be passed on to companies or third parties. Data collection is carried out in accordance with the procedure list 10-15, supervised by the data protection officer at RWTH Aachen University.'
